# Pervaporative Dehydration of Organic Solvents Using High-Silica CHA-Type Zeolite Membrane

**DOI:** 10.3390/membranes11030229

**Published:** 2021-03-23

**Authors:** Yasuhisa Hasegawa, Chie Abe, Ayumi Ikeda

**Affiliations:** National Institute of Advanced Industrial Science and Technology (AIST), Research Institute of Chemical Process Technology, 4-2-1 Nigatake, Sendai 983-8551, Japan; abe-chie@aist.go.jp (C.A.); a-ikeda@aist.go.jp (A.I.)

**Keywords:** zeolite membrane, high-silica CHA-type zeolite, dehydration, pervaporation, separation

## Abstract

A high-silica chabazite (CHA) type zeolite membrane was prepared on the porous α-Al_2_O_3_ support tube by the secondary growth of seed particles. The dehydration performances of the membrane were determined using methanol, ethanol, 2-propanol, acetone, acetic acid, methyl ethyl ketone (MEK), tetrahydrofuran (THF), *N,N*-dimethylformamide (DMF), dimethyl sulfoxide (DMSO), and *N*-methyl-2-pyrolidone (NMP) at 303–373 K. As a result, the dehydration performances of the membrane were categorized to following three types: (1) 2-propanol, acetone, THF, and MEK; (2) ethanol and acetic acid; and (3) methanol, DMF, and DMSO, and NMP. The adsorption isotherms of water, methanol, ethanol, and 2-propanol were determined to discuss the influences of the organic solvents on the permeation and separation performances of the membrane. For 2-propanol, acetone, MEK, and THF solutions, the high permeation fluxes and separation factors were obtained because of the preferential adsorption of water due to molecular sieving. In contrast, the permeation fluxes and separation factors were relatively low for methanol, DMF, and DMSO, and NMP solutions. The lower dehydration performance for the methanol solution was due to the adsorption of methanol. The permeation fluxes for ethanol and acetic acid solution were ca. 1 kg m^−2^ h^−1^. The significantly low flux was attributed to the similar molecular diameter to the micropore size of CHA-type zeolite.

## 1. Introduction

Membrane separation has attracted much attention as an energy-saving separation technology. In particular, membrane dehydration is a separation process with a great energy saving effect, since water forms an azeotrope with various organic solvents easily. It is well known that NaA-type zeolite membrane has been used for the dehydration of biomass-derived ethanol [1,2,3]. However, the NaA-type zeolite membrane cannot be applied to various processes because of its low acid resistance [4].

We could form polycrystalline chabazite (CHA) type zeolite layers with various Si/Al ratios on porous α-Al_2_O_3_ tubes previously [5,6,7,8,9,10]. In particular, the membrane with Si/Al = 18 showed the extremely high acid resistance [10]. The membrane was stable for more than three weeks in a 20wt% sulfuric acid solution, and in addition, it was possible to dehydrate the 75wt% sulfuric acid solution. However, the dehydration properties of the high-silica CHA-type zeolite membrane have not been clear for various organic solvents containing water. Moreover, there are few studies about the permeation behaviors of zeolite membranes for dehydration in detail, although that for the gas permeation was studied by several groups in the 1990s [11,12,13,14,15].

In this paper, we present the dehydration performances of the high-silica CHA-type zeolite membrane that were determined for several organic solvents. Furthermore, the permeation and separation behaviors of the membrane are discussed in this manuscript.

## 2. Experimental

### 2.1. Membrane Preparation

A CHA-type zeolite membrane was prepared by the secondary growth of seed particles [9,10]. The seed particles were synthesized using sodium aluminate (FUJIFILM Wako), sodium hydroxide (FUJIFILM Wako)*, N*,*N*,*N*-trimethyl-1-adamantammonium hydroxide solution (SDA, 25 wt%, Sachem), and ultrastable Y-type zeolite particles (Tosoh Corp., HSZ-390HUA). The molar composition of the synthesis solution was 40 SiO_2_: 1 Al_2_O_3_: 4 Na_2_O: 8 SDA: 800 H_2_O. The mixture was poured into a Teflon-lined stainless-steel autoclave after stirring for 1 h, and a hydrothermal reaction was carried out at 433 K for four days. The particles were recovered by filtration, washed with distilled water, and dried in an oven at 383 K overnight to obtain the seed particles.

For the membrane formation, a synthesis solution was prepared by the same manner as that for the seed particles, and the molar composition of the solution was 45 SiO_2_:1 Al_2_O_3_:4.5 Na_2_O:3.4 SDA:4500 H_2_O. The porous α-Al_2_O_3_ tube was used as the support, and the properties were as follows: outer diameter = 2.0 mm; inner diameter = 1.5 mm; mean pore diameter = 0.30 μm; and porosity = 50%. The outer surface of the support tube was rubbed with the seed particles to implant seeds for nucleation, and the tube was added to the autoclave filled with 30 g of the synthesis solution. The autoclave was placed in the oven at 433 K for 18 h horizontally to form a polycrystalline CHA-type zeolite layer. The support tube was recovered from the autoclave after cooling to room temperature, and the membrane was washed with distilled water several times and dried overnight at ambient temperature. Finally, the membrane was calcined in air at 773 K for 10 h to burn out the structure-directing agent.

### 2.2. Characterization

The morphology was observed by a scanning electron microscope (TM-1000, Hitachi High-technologies, Tokyo, Japan), and the composition was analyzed using an energy-dispersive X-ray analyzer attached to the SEM. The crystal structure was identified by X-ray diffraction (Smart-Lab, Rigaku, Tokyo, Japan). The amount of adsorbed water, methanol, ethanol, or 2-propanol vapors on the CHA-type zeolite particles were determined using a vapor adsorption unit with a constant volume cell (Belsorp max, MicrotracBEL, Tokyo, Japan). The seed particles were calcined under the same conditions as that for the membrane were used for the vapor adsorption experiments. The particles were pretreated at 423 K overnight before each measurement.

### 2.3. Pervaporation Experiments

**Figure 1:**
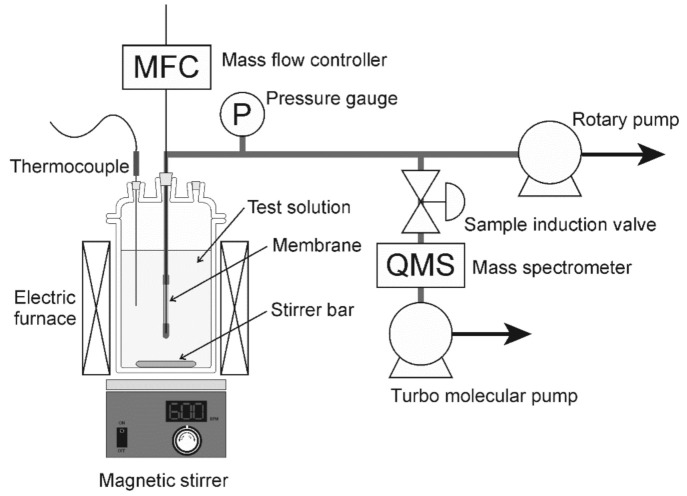
Schematic illustration of pervaporation unit used in this study [16].

Figure 1 shows the schematic illustration of a pervaporation unit [16]. Either methanol, ethanol, 2-propanol, acetone, methyl ethyl ketone (MEK), tetrahydrofuran (THF), *N,N*-dimethylformamide (DMF), dimethyl sulfoxide (DMSO), or *N*-methyl-2-pyrolidone (NMP) were used as the test solvents in this study. One end of the membrane was connected to a stainless-steel tube using epoxy resin (GL Science, Torr Seal), and the other end was capped. The effective membrane area for permeation was 1.2 cm^2^. The membrane was added to the organic solvents containing water. Helium was introduced into the inner surface of the membrane through a capillary tube, and, furthermore, the inner surface of the membrane was evacuated by a rotary pump below 1 kPa. The total pressure of the solution was kept at atmospheric pressure. The gas composition in the evacuated stream was analyzed using a mass spectrometer (HAL-301RC, Hiden Analytical, London, UK). The total pressure in the chamber of the mass spectrometer, electron impact energy, and emission were 10^−6^ Pa, 70 eV, and 30 μA, respectively. It is well known that some fragments were formed under the operating condition of the mass spectrometer. For example, ethanol exhibits peaks at the mass-to-charge ratios (*m/z*) of 26−31, 42, 43, 45, and 46. In this study, the following *m/z* values of the highest signal were selected for detecting the respective components: helium (*m/z* = 4), water (*m/z* = 18), methanol (*m/z* = 31), ethanol (*m/z* = 31), 2-propanol (*m/z* = 45), acetone (*m/z* = 43), MEK (*m/z* = 28), THF (*m/z* = 42), DMF (*m/z* = 73), DMSO (*m/z* = 63), and NMP (*m/z* = 44). The analytical accuracy of the mass spectrometer was less than 3% in the experimental setup. The permeation flux of component *i*, *J_i_*, was calculated as follows [16]:

(1) $$J_{i}=\frac{N_{\mathrm{He}}}{S}\cdot \frac{y_{i}}{y_{\mathrm{He}}},$$

where *N*_He_, *S*, and *y_i_* are the molar flow rate of helium, effective membrane area for permeation, and mole fraction of component *i* in the evacuated stream, respectively. For mixtures, the overall permeation flux *J*_t_ was calculated as follows:

(2) $$J_{t}=3600\sum_{i}J_{i}M_{i},$$

where *M_i_* is the molecular weight of component *i*. The separation factor of water with respect to organic solvents *α*_w/o_ was defined as follows:

(3) $$\alpha_{w/o}=\frac{\left(y_{w}/y_{o}\right)}{\left(x_{w}/x_{o}\right)},$$

where *x_i_* is the mole fraction of component *i* in the test solution.

The permeance of component *i*, *Q_i_*, was calculated by dividing the permeation flux by the partial pressure differences across the membrane as follows [17]:

(4) $$Q_{i}=\frac{J_{i}}{p_{f,i}-p_{p,i}},$$

where *p*_f,*i*_ and *p*_p,*i*_ are the partial vapor pressures of component *i* in the feed solution and the evacuated stream, respectively. The partial vapor pressure in the feed can be estimated using the Wilson constants and Antoine constants, as listed in Table 1 [18]. The vapor-liquid equilibrium for binary mixtures is described as follows [18]:

(5) $$z_{i}p_{t}=x_{i}\gamma_{i}p_{i}^{},$$

where *p*_t_ is the total vapor pressure. *z_i_*, *p_i_*, *γ_i_* are the mole fraction of component *i* in the vapor phase, vapor pressure of component *i*, and activity coefficient of component *i*, respectively. The total vapor pressure is calculated as follows:

(6) $$p_{t}=x_{i}\gamma_{i}p_{i}+x_{j}\gamma_{j}p_{j},$$

## 3. Results and Discussion

### 3.1. Characterization

**Figure 2:**
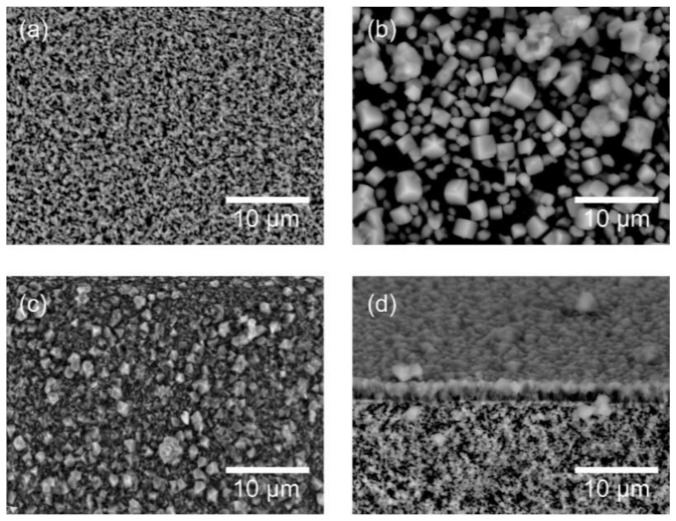
SEM images of (**a**) porous support tube, (**b**) seed particles, and (**c**) top surface and (**d**) fractured section of the membrane.

**Figure 3:**
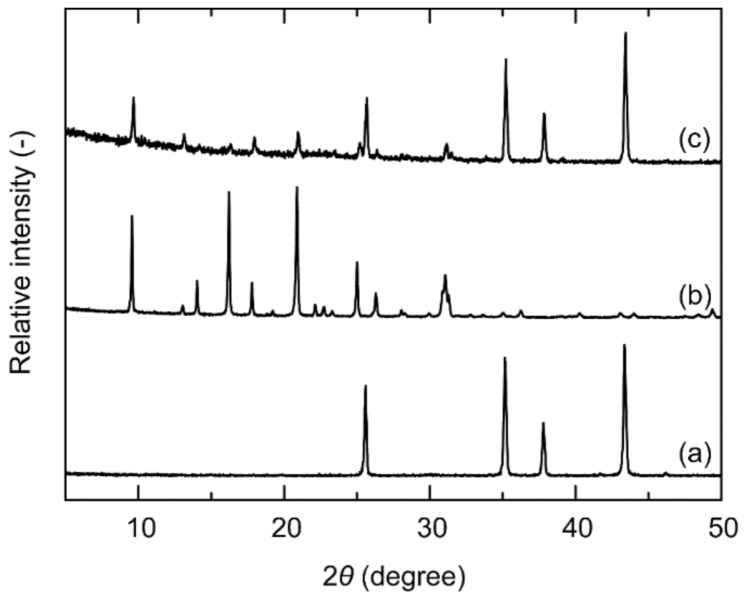
XRD patterns of (**a**) support tube, (**b**) seed particles, and (**c**) membrane.

Figure 2 shows the SEM images of the support tube, seed particles, and membrane. The shape of the seed particles was cubic, and the sizes were 1–5 μm. As shown in Figure 3b, the seed particles gave the diffraction peaks due to (1 1 1), (1 1 −1), (2 0 −1) faces of the CHA-type zeolite at 2*θ* = 9.6°, 16.2°, and 20.9°, respectively [19]. No peaks due to the other crystals were appeared. The outer surface of the porous support tube was covered with a polycrystalline layer (thickness = 2–3 μm, Si/Al = 17), as shown in Figure 2c. The XRD pattern of the membrane contained both the peaks of the support tube and seed particles, as shown in Figure 3c. These results suggest that the polycrystalline CHA-type zeolite layer could be formed on the porous α-Al_2_O_3_ tube in this study.

**Figure 4:**
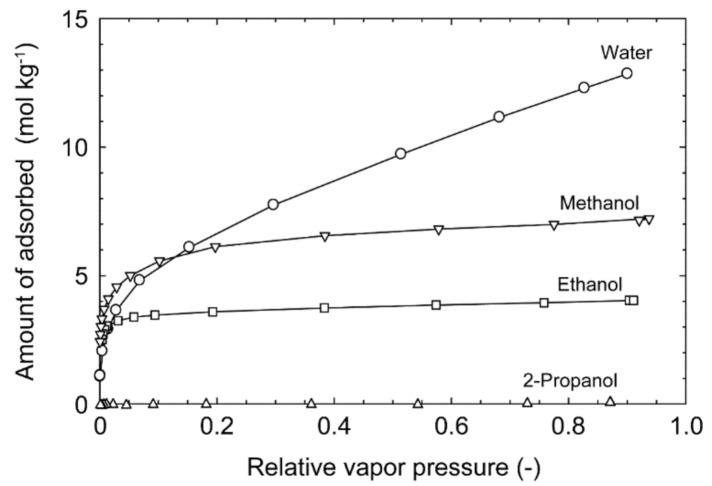
Adsorption isotherms of water, methanol, ethanol, and 2-propanol at 303 K.

Figure 4 shows the adsorption isotherms of water, methanol, ethanol, and 2-propanol at 303 K. The amounts of adsorbed 2-propanol were marginal, while the amounts of adsorbed water, methanol and ethanol were 12.8, 7.1, and 4.0 mol kg^−1^ around the relative vapor pressure of 0.9, respectively. This indicates that the channel diameter of the CHA-type zeolite (0.38 nm) was smaller than the molecular size of 2-propanol and larger than diameters of water, methanol, and ethanol molecules [20]. Moreover, the slope of the isotherms for methanol and ethanol were higher than that of water at low pressures.

### 3.2. Dehydration Performances

Table 2 shows the dehydration performances of the high-silica CHA-type zeolite membrane for several organic solvents. The dehydration performances for methanol, acetone and THF were determined at boiling temperatures of their solutions. For a 90wt% 2-propanol solution at 348 K, the overall permeation flux and separation factor were 10.0 kg m^−2^ h^−1^ and 82,200, respectively. The CHA-type zeolite membrane with Si/Al = 3 showed the extremely high dehydration performances (*J*_t_ = 19.0 kg m^−2^ h^−1^ and *a*_w/o_ > 100,000) because of the hydrophilic properties due to the high aluminum content [7]. Sato et al. developed commercially available CHA-type zeolite membrane with Si/Al = 7 [21]. Since the thickness of their membrane was ca. 10 μm, the flux was lower than that of our membrane. Imasaka et al. also developed the high-silica CHA-type zeolite membranes with Si/Al = 11, and their membrane showed the higher permeation flux according to the lower permeation resistance of the support [22]. Compared to these previous reports about the CHA-type zeolite membranes, our membrane showed the relatively high permeation flux and separation factor for 2-propanol, acetone, THF, and MEK.

The dehydration performances listed in Table 2 could be categorized to three types by organic solvents as follows:

(I) 2-propanol, acetone, THF, and MEK: high fluxes and high separation factors;
(II) ethanol and acetic acid: low fluxes and high separation factors;
(III) methanol, DMF, and DMSO, and NMP: low fluxes and low separation factors.

For DMF, DMSO, and NMP, the boiling points are higher than those of the other solvents, as listed in Table 3 [24]. The lower fluxes for these solutions are according to the lower vapor pressures due to the higher boiling points [25]. Moreover, the high dipole moments of DMF, DMSO, and NMP maybe influence the separation performance.

### 3.3. Evaluation of Permeation Behavior

**Figure 5:**
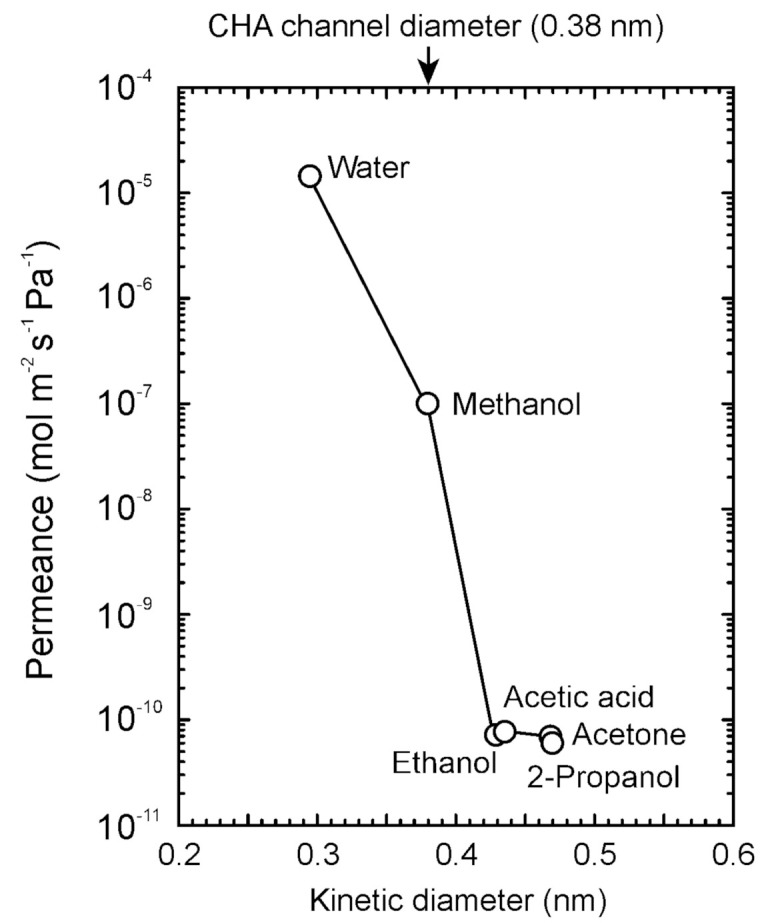
Influence of the molecular size on the permeances of single component solvents at 303 K.

The pervaporation experiments were carried out using methanol, ethanol, 2-propanol, acetone, and acetic acid to discuss the influence of organic solvent. First, the permeation properties of pure solvent were determined. Figure 5 shows the influence of molecular size on the single component permeances at 303 K. The permeance was 1.45 × 10^−5^ mol m^−2^ s^−1^ Pa^−1^ for water (diameter = 0.2955 nm), decreased with the molecular size, and reached 7.27 × 10^−11^ mol m^−2^ s^−1^ Pa^−1^ for ethanol (diameter = 0.4299 nm). The relationship between the molecular size and the permeance indicates that molecules pass through the micropores of CHA-type zeolite. However, 2-propanol was detected in the permeate stream (permeance = 6.07 × 10^−11^ mol m^−2^ s^−1^ Pa^−1^), although 2-propanol could not adsorb on the CHA-type zeolite, as shown in Figure 4. This proposes that the CHA-type zeolite membrane has a little small pinhole.

The effective diffusion coefficients of water, methanol, and ethanol can be estimated using the adsorption isotherms in Figure 4 and the permeances in Figure 5 as follows [14,15]:

(7) $$Q_{i}=\frac{\rho D_{i}(q_{f,i}-q_{p,i})}{\delta (p_{f,i}-p_{p,i})},$$

where, *q_i_*, *K_i_*, and *D_i_* are the membrane thickness, amount of adsorbed, Langmuir constant, and diffusion coefficient, respectively. The diffusion coefficients of water, methanol, and ethanol were 1.0 × 10^−11^, 1.4 × 10^−12^, and 1.0 × 10^−15^ m^2^ s^−1^, respectively. Barrer et al. determined the diffusion coefficients of water in several zeolites, and that was 1.3 × 10^−11^ m^2^ s^−1^ at 318 K for CHA-type zeolite [26]. The similar value means that water molecules diffused the intracrystalline micropores of CHA-type zeolite.

**Figure 6:**
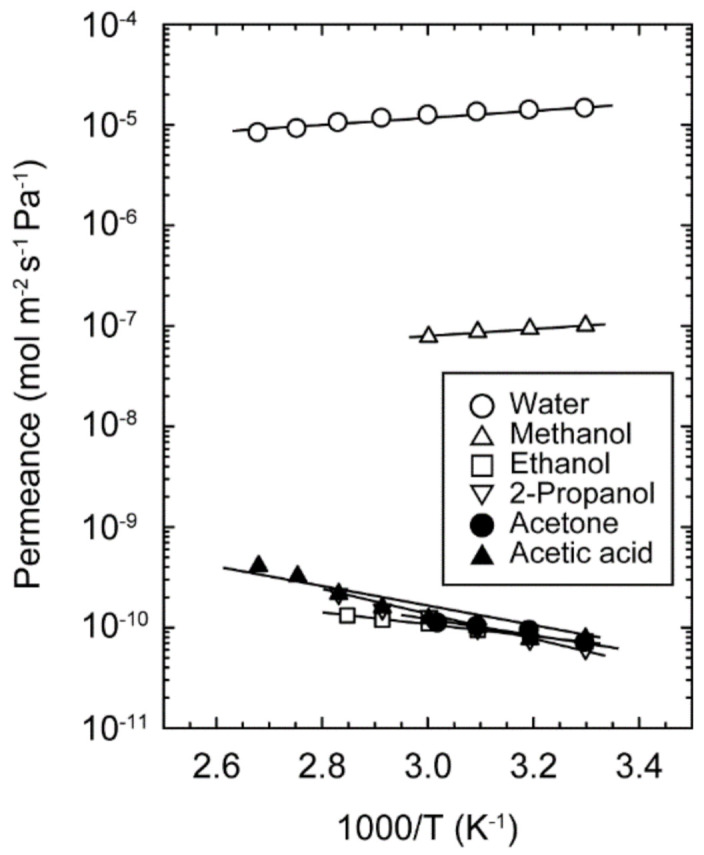
Effect of temperatures on the permeances of single component water, methanol, ethanol, acetic acid, acetone, and 2-propanol.

Figure 6 shows the effect of temperatures on the permeances of the single component solvents. The permeances of water and methanol decreased with temperature, while those of ethanol, 2-propanol, acetone, and acetic acid showed reverse trends. It is well known that the permeance decreases with temperature when the molecules are transferred by the surface diffusion [12,15]. Therefore, these results indicate that the adsorbed water and methanol molecules diffused within the micropores of the CHA-type zeolite. When the effect of diffusion is greater than adsorption, the permeance increases with temperature. Although ethanol adsorbed on CHA-type zeolite, as shown in Figure 4, the permeance increased with temperature. This suggests that the activation energy to overcome the potential barrier from pore walls is needed for moving within the zeolite micropores. Accordingly, ethanol molecules can adsorb on CHA-type zeolite, while it is difficult to diffuse within the micropores.

**Figure 7:**
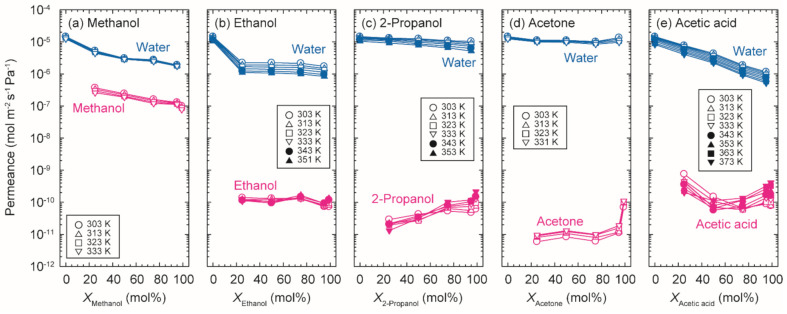
Influences of the concentration of organic solvents in the feed solutions on the permeances of water and organic solvents at 303–373 K.

Figure 7 shows the influences of the organic solvent concentrations on the permeances of water and organics for binary mixtures of water/methanol, water/ethanol, water/2-propanol, water/acetone, and water/acetic acid at 303–373 K. The permeances of water and methanol for the methanol solution decreased as the methanol concentration increased. Krishna et al. simulated the adsorption isotherms and self-diffusivities for water–alcohol mixtures by configurational-bias Monte Carlo and molecular dynamics simulations [27,28]. Water and alcohol molecules interact and form strong hydrogen bonding in the zeolite channels, and both the diffusion coefficients of water and methanol decrease with increasing the methanol occupancy on the CHA-type zeolite. The reduction of permeances for the methanol solutions were attributed to the reduction of diffusion coefficients by the hydrogen bonding between water and methanol. The similar trends were observed for the ethanol solutions and acetic acid solution. For the ethanol solutions, in particular, the water permeance decreased significantly at the ethanol concentration of 25 mol% and was almost constant at the higher ethanol concentrations. It is considered that the ethanol molecules located in the zeolite micropores inhibited the permeation of water. For 2-propanol, which cannot penetrate into the zeolite micropores, the influence of the 2-propanol concentration on the permeances of water were marginal. The 2-propanol permeances increased with increasing the concentration because of the easy penetration of 2-propanol molecules into the pinholes. The similar tendencies were found for acetone.

**Figure 8:**
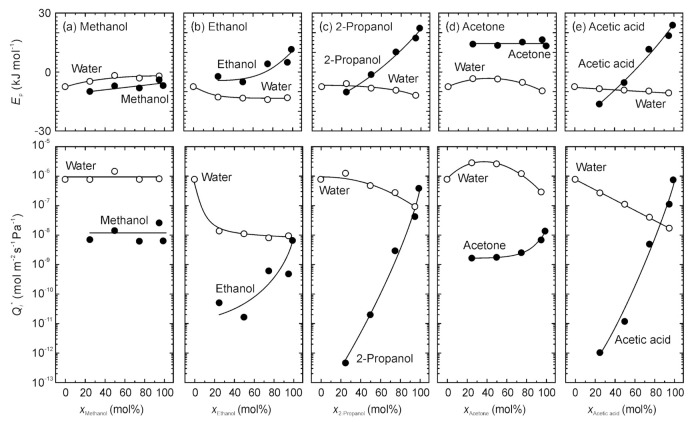
Pre-exponential factors and activation energies of water, methanol, ethanol, 2-propanol, acetone, and acetic acid as function of the organic solvent concentrations.

The effect of temperatures on the permeation is described using the Arrhenius equation:

(8) $$Q_{i}=Q_{i}^{*}\exp\left(-\frac{E_{p}}{RT}\right),$$

where Q_i_^*^ and *E*_p_ are the pre-exponential factor and activation energy for permeation, respectively. Figure 8 shows the relationship between the pre-exponential factors and activation energies for permeation through the high-silica CHA-type zeolite membrane. The pre-exponential factors and activation energies for the ethanol, 2-propanol, and acetic acid increased with increasing their concentration, while those of water showed the reverse tendency. For methanol, in contrast, the pre-exponential factors were independent of the methanol concentration, and the activation energies increased.

**Figure 9:**
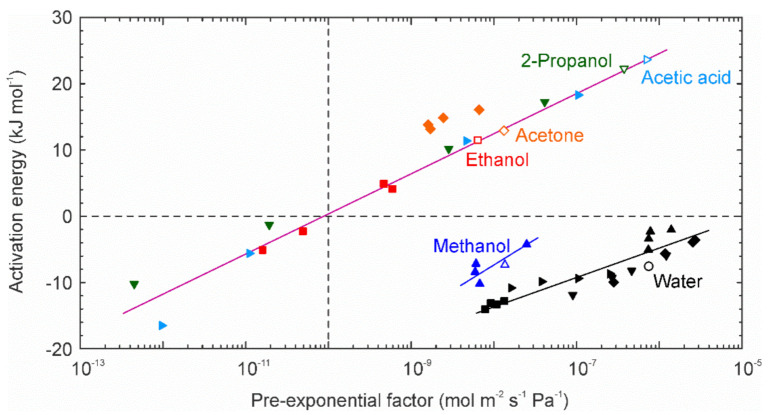
Relationship between pre-exponential factors and activation energies for permeation through the high-silica CHA-type zeolite membrane. (Open key) pure components and (closed key) mixtures.

Figure 9 represents the relationship between the pre-exponential factors and activation energies. The experimental data could be plotted on three lines, such as water, methanol, and the other solvents. The pre-exponential factor and activation energy of pure water were 7.6 × 10^−7^ mol m^−2^ s^−1^ Pa^−1^ and −7.6 kJ mol^−1^, respectively. Even if mixing with organic solvents, the activation energy was negative, and the pre-exponential factors ranged more than 10^−9^ mol m^−2^ s^−1^ Pa^−1^. Methanol showed the similar trend, although the activation energy was slightly large. The activation energies of pure ethanol, 2-propanol, acetone, and acetic acid were higher than 10 kJ mol^−1^. However, those were lower than 0 kJ mol^−1^ at the concentrations below 50 mol%. The pre-exponential factors also decreased to less than 10^‒10^ mol m^−2^ s^−1^ Pa^−1^.

Morooka and coworkers proposed the adsorption-diffusion model to discuss the influence of the adsorption and diffusion on the permeation properties of zeolite membranes. The permeance can be described using the adsorption coefficient *S_i_* and diffusion coefficient *D_i_* as follows:

(9) $$Q_{i}\delta =S_{i}D_{i}.$$

Since the effect of temperatures on the adsorption coefficients and diffusion coefficients can also expressed by the Arrhenius equation, Equation (11) can be converted to

(10) $$Q_{i}^{*}\exp\left(-\frac{E_{p}}{RT}\right)=\frac{S_{i}^{*}D_{i}^{*}}{\delta }\exp\left(-\frac{E_{D}-\Delta H_{a}}{RT}\right).$$

The activation energy for permeation is equal to the difference between the activation energy for diffusion (*E*_D_) and the heat of adsorption (−Δ*H*_a_). As described above, for water and methanol, the activation energies for permeation were negative. Therefore, those molecules are transferred by the surface diffusion. On the contrary, for pure ethanol, 2-propanol, acetone, and acetic acid, the activation energies for permeation were positive. This indicates that those molecules permeated through the membrane by the activation diffusion. When the concentrations of these solvents are below 50 mol%, water molecules adsorb on the micropores of CHA-type zeolite. Therefore, the adsorption coefficients of organic solvents are significantly low. Since the organic molecules moved with water molecules under those conditions by the hydrogen bonding, the activation energies will reduce to the same level as that of water.

## 4. Conclusions

The polycrystalline CHA-type zeolite layer with Si/Al = 17 could be formed on the outer surface of the porous α-Al_2_O_3_ tube by the secondary growth of seed particles. The dehydration performances of the membrane were determined using methanol, ethanol, 2-propanol, acetone, acetic acid, MEK, THF, DMF, DMSO, and NMP at 303–373 K. The membrane showed the high permeation fluxes and separation factors for 2-propanol, acetone, MEK, and THF. For example, for the 90 wt% 2-propanol solution at 348 K, the overall permeation flux and separation factor were 10.0 kg m^−2^ h^−1^ and 82,200, respectively. The high dehydration performances were attributed to the selective adsorption of water by molecular sieving. In contrast, the permeation fluxes and separation factors were relatively low for methanol, DMF, DMSO, and NMP. The lower performances for the methanol solutions were according to the surface diffusion of methanol. Moreover, for ethanol and acetic acid, the separation factors were more than several thousands, although the permeation fluxes were low (ca. 1 kg m^−2^ h^−1^). Those molecules hindered water permeation by their similar molecular size to the micropore diameter.

## Figures and Tables

**Table 1:** Wilson constants and Antoine constants for organic solvents containing water [18].

Solvents	Wilson Constants		Antoine Constants		
	Λ_wo_	Λ_ow_	*A*	*B*	*C*
Water	-----	-----	8.02754	1705.616	231.405
Methanol	0.89781	0.55148	8.07919	1581.34	239.65
Ethanol	0.79133	0.21618	8.04494	1554.3	222.65
2-Propanol	0.77714	0.04857	6.6604	813.055	132.93
Acetic acid	0.23965	1.67589	7.18807	1416.7	211
Acetone	0.42161	0.15813	7.29958	1312.25	240.705

**Table 2:** Dehydration performances of the CHA-type zeolite membrane for several organic solvents.

Si/Al (-)	Solvent	Water Content (wt%)	Temp (K)	*J*_t_ (kg m^−2^ h^−1^)	*α*_w/o_ (-)	Ref.
17	Methanol	10	333	2.0	7	This work
	Ethanol	10	348	1.2	5400	
	Acetic acid	10	348	0.9	24,500	
	2-Propanol	10	348	10.0	82,200	
	Acetone	10	323	4.9	>100,000	
	THF	10	338	8.9	>100,000	
	MEK	10	348	15.5	10,800	
	DMF	10	348	2.6	2000	
	DMSO	10	348	1.6	1860	
	NMP	10	348	4.0	565	
3	Methanol	15	330	5.4	680	[7]
	Ethanol	10	350	14.5	15,000	
	2-Propanol	10	350	19.0	>100,000	
7	2-Propanol	10	348	6.4	1600	[21]
	NMP	10	363	4.5	640	
11	2-Propanol	20	348	20.0	1130	[22]
18	Acetic acid	50	348	7.9	2,500	[23]

**Table 3:** Chemical properties of organic solvents [20].

Solvent	Formula	*M_i_* (×10^3^ kg mol^−1^)	*b.p.* (K)	Kinetic Diameter (nm)	Dipole Moment (Debye)
Methanol	CH_4_O	32.04	338	0.3803	1.71
Ethanol	C_2_H_6_O	46.07	352	0.4299	1.73
Acetic acid	C_2_H_4_O_2_	60.05	391	0.4356	1.74
2-Propanol	C_3_H_8_O	60.10	355	0.4699	1.66
Acetone	C_3_H_6_O	58.08	329	0.4691	2.88
THF	C_4_H_8_O	72.11	339	0.4856	1.63
MEK	C_4_H_8_O	72.11	353	0.5036	2.80
DMF	C_3_H_7_NO	73.09	426	0.582 ^1^	3.86
DMSO	C_2_H_6_SO	78.13	462	0.651 ^1^	4.30
NMP	C_5_H_9_NO	99.13	475	0.512 ^1^	3.59

^1^ The diameters are estimated *σ* = (1.585*V*_b_/(1 + 1.3*d*^2^))^1/3^, and the molar volume at normal boiling point *V*_b_ is calculated by *V*_b_ = *V*_c_(0.29056 − 0.08775ω)*^f^*, where *f* is defined by (1 − *T*_b_/*T*_c_)^2/7^ [24].

## Data Availability

Not applicable.

## Nomenclature

*A*	Antoine constant (dimensionless)
*B*	Antoine constant (dimensionless)
*C*	Antoine constant (dimensionless)
*D_i_*	diffusion coefficient of component *i* (m^2^ s^−1^)
*D_i_^*^*	diffusion coefficient of component *i* at infinite temperature (m^2^ s^−1^)
*E*_D_	activation energy for diffusion (kJ mol^−1^)
*E*_p_	activation energy for permeation (kJ mol^−1^)
−Δ*H*_a_	heat of adsorption (kJ mol^−1^)
*J_i_*	permeation flux of component *i* (mol m^−2^ s^−1^)
*J_t_*	overall permeation flux (kg m^−2^ h^−1^)
*M_i_*	molecular weight (kg mol^−1^)
*N_He_*	molar flow rate of helium (mol s^−1^)
*p_i_*	partial pressure of component *i* (Pa)
*p_t_*	total vapor pressure (Pa)
*q_i_*	amount of adsorbed component *i* (mol kg^−1^)
*Q_i_*	permeance (mol m^−2^ s^−1^ Pa^−1^)
*Q_i_^*^*	permeance at infinite temperature (mol m^−2^ s^−1^ Pa^−1^)
*S*	effective membrane area for permeation (m^2^)
*S_i_*	adsorption coefficient of component *i* (mol m^−3^ Pa^−1^)
*S_i_^*^*	adsorption coefficient of component *i* at infinite temperature (mol m^−3^ Pa^−1^)
*T*_b_	boiling temperature (K)
*T*_c_	critical temperature (K)
*V*_b_	molar volume at boiling temperature (cm^3^ mol^−1^)
*V*_c_	molar volume at critical temperature (cm^3^ mol^−1^)
*x_i_*	mole fraction of component *i* in solution (dimensionless)
*y_i_*	mole fraction of component *i* in the evacuated stream (dimensionless)
*z_i_*	mole fraction of component *i* in vapor phase (dimensionless)
*Symbols*	
*α*_w/o_	separation factor of water with respect to organic solvents (dimensionless)
*δ*	membrane thickness (m)
*γ_i_*	activity coefficient of component *i* (dimensionless)
Λ*_ij_*	Wilson parameter of components *i-j* (dimensionless)
*ρ*	density of zeolite (kg m^−3^)
*ω*	acentric factor (dimensionless)
Subscripts	
f	feed solution
p	permeate side
o	organic solvent
w	water
